# Protocol of the Italian Radical Cystectomy Registry (RIC): a non-randomized, 24-month, multicenter study comparing robotic-assisted, laparoscopic, and open surgery for radical cystectomy in bladder cancer

**DOI:** 10.1186/s12885-020-07748-7

**Published:** 2021-01-11

**Authors:** Angelo Porreca, Katie Palmer, Walter Artibani, Alessandro Antonelli, Lorenzo Bianchi, Eugenio Brunocilla, Aldo Massimo Bocciardi, Maurizio Brausi, Gian Maria Busetto, Marco Carini, Giuseppe Carrieri, Antonio Celia, Luca Cindolo, Giovanni Cochetti, Renzo Colombo, Ettore De Berardinis, Ottavio De Cobelli, Fabrizio Di Maida, Amelio Ercolino, Franco Gaboardi, Antonio Galfano, Andrea Gallina, Michele Gallucci, Carlo Introini, Ettore Mearini, Andrea Minervini, Francesco Montorsi, Gennaro Musi, Giovannalberto Pini, Riccardo Schiavina, Silvia Secco, Sergio Serni, Claudio Simeone, Giovanni Tasso, Daniele D’Agostino

**Affiliations:** 1 Istituto Oncologico Veneto IRCCS, Padova, Italy; 2 Department of Internal Medicine and Geriatrics, Università Cattolica del Sacro Cuore, Largo Francesco Vito, 1, 00168, 00136 Rome, Italy; 3 Department of Urology, Policlinico Abano Terme, Abano Terme, PD Italy; 4 Department of Urology, Azienda Ospedaliera Universitaria Integrata (A.O.U.I.), Verona, Italy; 5 Department of Urology, University of Bologna, Bologna, Italy; 6 Division of Urology, IRCCS Azienda Ospedaliero-Universitaria di Bologna, Bologna, Italy; 7 Struttura Complessa Urologia, ASST, Grande Ospedale Metropolitano Niguarda, Milan, Italy; 8 Divisione Urologia AUSL, Modena, Italy; 9 Department of Maternal-Child and Urological Sciences, Sapienza Rome University, Policlinico Umberto I Hospital, Rome, Italy; 10 Department of Urology, Careggi Hospital, University of Florence, Florence, Italy; 11 Unit of Oncologic Minimally-Invasive Urology and Andrology, Careggi Hospital, Florence, Italy; 12 Urology and Renal Transplantation Unit, Department of Medical and Surgical Sciences, University of Foggia, Foggia, Italy; 13 Department of Urology, San Bassiano Hospital, Bassano Del Grappa, Italy; 14 Department of Urology, “Villa Stuart” Private Hospital, Rome, Italy; 15 Department of Urology, University of Perugia, Perugia, Italy; 16 Department of Urology and Division of Experimental Oncology, URI, Urological Research Institute, IRCCS San Raffaele Scientific Institute, Milan, Italy; 17 IEO European Institute of Oncology, IRCCS, Milan, Italy; 18 Department of Hematology and Hemato-Oncology, Universty of Milan, Milan, Italy; 19 Department of Urology, San Raffaele Turro Hospital, Milano, Italy; 20 Department of Urology, E.O. Ospedali Galliera, Genova, Italy; 21 Department of Urology, University of Brescia, Brescia, Italy

**Keywords:** Cancer, Neoplasm; bladder, Urinary, Robotic, Surgery, Bladder reconstruction, Prostate

## Abstract

**Background:**

Bladder cancer is the ninth most common type of cancer worldwide. In the past, radical cystectomy via open surgery has been considered the gold-standard treatment for muscle invasive bladder cancer. However, in recent years there has been a progressive increase in the use of robot-assisted laparoscopic radical cystectomy. The aim of the current project is to investigate the surgical, oncological, and functional outcomes of patients with bladder cancer who undergo radical cystectomy comparing three different surgical techniques (robotic-assisted, laparoscopic, and open surgery). Pre-, peri- and post-operative factors will be examined, and participants will be followed for a period of up to 24 months to identify risks of mortality, oncological outcomes, hospital readmission, sexual performance, and continence.

**Methods:**

We describe a protocol for an observational, prospective, multicenter, cohort study to assess patients affected by bladder neoplasms undergoing radical cystectomy and urinary diversion. The Italian Radical Cystectomy Registry is an electronic registry to prospectively collect the data of patients undergoing radical cystectomy conducted with any technique (open, laparoscopic, robotic-assisted). Twenty-eight urology departments across Italy will provide data for the study, with the recruitment phase between 1st January 2017-31st October 2020. Information is collected from the patients at the moment of surgical intervention and during follow-up (3, 6, 12, and 24 months after radical cystectomy). Peri-operative variables include surgery time, type of urinary diversion, conversion to open surgery, bleeding, nerve sparing and lymphadenectomy. Follow-up data collection includes histological information (e.g., post-op staging, grading, and tumor histology), short- and long-term outcomes (e.g., mortality, post-op complications, hospital readmission, sexual potency, continence etc).

**Discussion:**

The current protocol aims to contribute additional data to the field concerning the short- and long-term outcomes of three different radical cystectomy surgical techniques for patients with bladder cancer, including open, laparoscopic, and robot-assisted. This is a comparative-effectiveness trial that takes into account a complex range of factors and decision making by both physicians and patients that affect their choice of surgical technique.

**Trial registration:**

ClinicalTrials.gov, NCT04228198. Registered 14th January 2020- Retrospectively registered.

## Background

### Epidemiology of bladder cancer

Bladder cancer is the ninth most common type of cancer worldwide, with 75% of the total burden occurring in men [1]. In Europe the age-standardized incidence rate is 19.1 for men and 4.0 for women, and mortality rates (per 100,000 persons years) are 3.2 for men and 0.9 for women [2]. Bladder cancer increases with age and, although it is higher in men than women [1], incidence in both sexes is expected to increase in European countries [3]. Cigarette smoking has been identified as one of the main risk factors for bladder cancer [3, 4], and incidence rates are higher in more developed countries [5].

At initial diagnosis, cancer neoplasms present as a non-muscle-invasive pathology in 70–75% of cases, and a muscle-invasive form in about a quarter of cases [6]. In about one third of patients with a diagnosis of muscle-invasive bladder tumor, the neoplasm is found to be a metastasis that was not identified during treatment of the primary tumor; in particular, lymph node involvement is identified during surgery in about 25% of patients receiving a radical cystectomy [7].

### Radical cystectomy: the gold standard

Since the 1960s, treatment with radical cystectomy has been the gold standard for invasive tumors of the bladder wall and is indicated for non-muscle-invasive bladder neoplasms with a high risk of progression or relapse that are non-responsive to intra-bladder immunotherapy (intravesical instillation of Bacillus Calmette-Guerin (BCG)), and in cases of pelvic neoplasms infiltrating the bladder [7–9]. In urology, cystectomy is considered to be one of the most technically challenging oncological interventions. Radical cystectomy involves removal of the entire bladder and lymph node dissection. In men, the prostate is often removed as well as the seminal vesicles, while in women the uterus, ovaries, and a small portion of the vagina and fallopian tubes are removed. Surgery includes the removal phase, followed by a reconstruction phase, which may consist of incontinent (using an ileal conduit) or continent (using a urethral or cutaneous neobladder) urinary diversion. The type of urinary diversion chosen varies according to the cancer stage and grade, prognosis, comorbidity, and functional status of the patient as well as potential contraindications to creating a neobladder [10, 11]. There are many factors that can be used to evaluate the success of treatment, including peri-operative outcomes (such as blood loss, mortality, hospital stay, complication rates etc), and short- or long-term outcomes (including overall survival, recurrence-free survival, and cancer-specific survival), as well as functional outcomes (such as continence and sexual potency).

### New, minimally invasive approaches and treatments: robot-assisted laparoscopic radical cystectomy

In the past, radical cystectomy via open surgery has been considered the gold-standard treatment for muscle invasive bladder cancer. However, in recent years there has been a progressive increase in the use of robot-assisted laparoscopic radical cystectomy [12], which is a challenging procedure suitable for both older and younger patients [13, 14] that can be improved with specific training and a skilled robotic team [15]. A recent review suggested that there are several advantages to the robot-assisted technique, such as lower blood loss and transfusion rate during surgery, and possibly faster gastrointestinal recovery, and a shorter length of hospital stay [16]. Studies have mostly found that peri-operative and short-term outcomes are similar when using robot-assisted versus open radical cystectomy [17–20], but some data suggests that assisted radical cystectomy improves minor, but not major, complications compared to open surgery [9], especially in terms of lower blood loss and shorter time to returning to a regular diet [17, 21, 22]. Few data on long-term oncological outcomes are available; initial studies report comparable outcomes for robot-assisted versus open radical cystectomy [19, 22–28]. Data on the long-term functioning of patients is also sparse, with no studies directly comparing urinary continence or sexual potency in patients receiving robot-assisted versus open radical cystectomy [16]. Robot-assisted radical cystectomy has been reported to cost more than open surgery due to higher supply costs [12], although to accurately assess the economic advantages of each technique, long-term studies that assess cancer recurrence, readmission rate, rehospitalization, and other health economic variables are needed.

### Need for large scale, multicenter registries

There are currently very few Randomized Control Trials (RCT) comparing robot-assisted laparoscopic versus open radical cystectomy [17], and previous non-randomized studies are hampered by small sample sizes and short follow-ups. Although RCTs are the preferred study design for evaluating treatment efficacy there is also a need for comparison-effective studies, because there are multiple factors that determine the choice and outcomes of surgical techniques in real-life clinical settings, including complex clinical decision making, patient and hospital characteristics, and surgical expertise. The current study aims to provide data to this rapidly developing field by creating a nationwide, multicenter registry with two-year post-surgery follow-up of bladder cancer patients who will undergo bladder cystectomy, with a comprehensive data collection on multiple outcomes.

## Aims

The aim of the project is to investigate the surgical, oncological, and functional outcomes of patients with bladder cancer who undergo radical cystectomy comparing three different surgical techniques (robotic-assisted, laparoscopic, and open surgery). Pre-, peri- and post-operative factors will be examined, and participants will be followed for a period of up to 24 months to identify risks of mortality, oncological outcomes, hospital readmission, sexual performance, and continence.

## Methods & design

### Study design and setting

This article describes the Italian Radical Cystectomy Registry - “Registro Italiano Cistectomia Radicale (RIC)”. The trial has been retrospectively registered on ClinicalTrials.gov on 14/01/2020 with reference number NCT04228198. The study is an observational, prospective, multicenter, cohort study to assess patients affected by bladder neoplasms undergoing radical cystectomy and urinary diversion. This protocol was developed in accordance with the Standard Protocol Items: Recommendations for Interventional Trials (SPIRIT) Statement. Twenty-eight participating centers across Italy will provide data for the study. All centers have a similar peri-operative pathway; all use enhanced recovery after surgery (ERAS) protocols. The centers are: Urology Clinic, University of Bologna; Department of Urology, AOU Careggi, Florence; European Institute of Oncology Milan; San Raffaele Hospital, Milan; University Hospital of Verona; Department of Urology, Policlinico Abano; Department of Urology, Spedali Civili, Brescia; Department of Urology and Kidney Transplantation, University of Foggia, Foggia; Galliera Hospital, Genova; ASST Niguarda Metropolitan Hospital, Niguarda; Policlinico Umberto I, Saproma; Department of Clinical Urology, University of Perugia; Department of Clinical Urology, AOUP Cisanello Hospital, Pisa; Department of Clinical Urology, Palermo University, Palermo; Department of Clinical Urology, Alessandria Hospital, Alessandria; Department of Clinical Urology, ASST Mantova, Mantova; Department of Clinical Urology, ASL Abruzzo; Department of Clinical Urology Ca Foncello Hospital, Treviso; Department of Clinical Urology II, Bari University, Bari; Department of Clinical Urology, Vittorio Emanuele Hospital, Catania; Department of Clinical Urology, Casa Sollievo della Sofferenza, Sgrotondo; Hospital Bassiano, Bassano; Department of Clinical Urology, Hospital San Francesco ASL 3, Nuoro; Department of Clinical Urology*,* Portogruaro; Department of Clinical Urology, Biella Hospital, Biella; Department of Clinical Urology Chioggia Hospital; Ausl Modena; Department of Urology and Kidney Transplantation, Bianchi-Melacrino-Morelli Grand Metropolitan Hospital.

### Participants

We will include patients with a histologically confirmed diagnosis of bladder cancer between January 1st 2017 and October 31st 2020, who will be treated in the participating medical centers. Eligibility screening will be conducted by the examining physician according to the following inclusion criteria: 1) male and female consecutively recruited patients; 2) age ≥ 18 years; 3) histologically confirmed diagnosis of bladder cancer eligible for radical cystectomy (according to EAU guidelines [7]) at date of enrollment; 4) providing written, informed consent. We aim to enroll approximately 1000 patients in the baseline data collection.

### Ethical issues

Data collection will be conducted in accordance with the *World Medical Association Declaration of Helsinki*. All potential participants will be required to sign an Informed Consent form. Ethical permission was received from the Ethical Committee of the University of Padova (number: 0042389). The standard of care will remain unchanged in patients participating in this study.

### Database design and management

The Italian Radical Cystectomy Registry is an electronic registry to prospectively collect the data of patients undergoing radical cystectomy conducted with any technique (open, laparoscopic, robotic-assisted), similar to one used by an International consortium. The protocol was designed by a Scientific Committee of Italian experts, who regularly reviews the database and proposes any relevant changes and integrations that may be useful for fulfilling the project aims. The database is based on an online platform. Information from patients will be collected in a confidential manner, according to Italian privacy Laws (Decreto Legislativo 196/2003); each patient will be identified via an anonymous identification code. Data will be used in an aggregated form. Each individual center will be responsible for the personal data collected in relation to the project. The information collected will be registered by the responsible physician in each center on the internet-based data storage file, which is accessible via username and password. The data will be regularly transferred to a global database. The data will be checked and cleaned by an Epidemiologist and any data inconsistencies or missing values will be directly resolved with the individual centers. All data will be analyzed anonymously. The Steering Committee will grant access for data analysis to individual researchers according to requests from the participating centers.

### Data collection

**Fig. 1:**
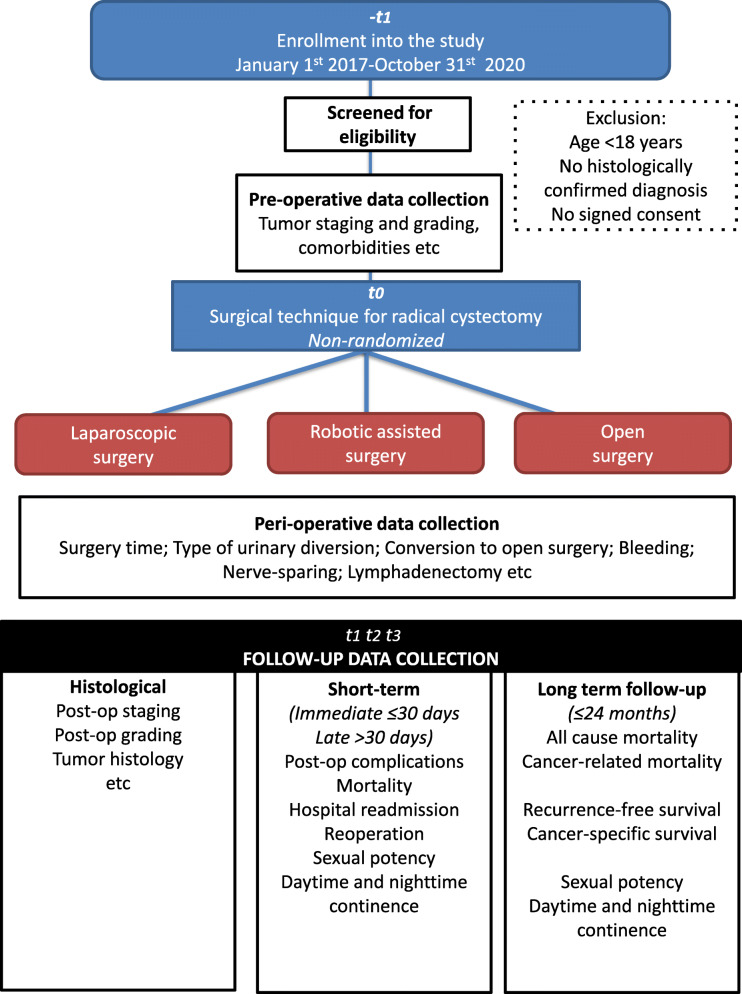
Study phases and summary of key data

Figure 1 illustrates the three phases of the study, and some of the key data that will be collected.

#### Phase I: recruitment of participating centers

All clinics and hospitals in Italy that currently carry out radical cystectomy with all three surgical techniques were contacted to establish interest in participating in the study. Participation is on a voluntary basis, and the centers will receive no additional funding for participating. A responsible physician in each participating center will be assigned the role of managing the data collection, guaranteeing the systematic recruitment of eligible patients, and supervising collection and coding of data according to the web-based data collection form. The questions used in the data collection form were designed by the Scientific and Steering Committee and include pre-specified categories for some questions and open fields for others, where appropriate. Shortly after the first date of patient recruitment the Scientific Committee reviewed a selection of the inputted patient data at each clinic to ensure homogeneity of reporting and modify any database issues.

#### Phase II: enrollment of patients

Urology departments of the participating centers will identify eligible patients during the recruitment phase (1st January 2017-31st October 2020) in accordance with the inclusion criteria. All potential participants will be given an informative letter explaining details of the study and will be asked to sign an informed consent form. After written, informed consent is provided information will be collected from the patients at the moment of surgical intervention and during follow-up (3, 6, 12, and 24 months after radical cystectomy). Surgical technique will not be randomized and will be assigned according to the clinical judgement of the surgeon. All participants will undergo usual clinical care.

#### Phase III: follow up

After 3, 6, 12, and 24 months post-surgery, data will be taken directly from the patients at their follow-up medical visits and entered into the online database. The physician identifying and recruiting patients will oversee the completion of the data collection forms during follow-up, even if the patient is subsequently treated in another center.

### Variables

Data will be collected by the medical team during the patient’s hospital admission and at clinical visits during the follow-up. Variables of interest include demographic variables (age, sex, etc), surgical factors (technique use, length of surgery, blood loss, type of urinary diversion, node burden, histological exams, surgical margins etc), mortality, morbidity (pre-, during- and post-surgery), oncological results, and functioning (continence and sexual performance) over follow-up, as detailed below. All health and biological data will be taken in accordance with usual clinical care of the patients and will be stored at the participating centers in accordance with national laws and guidelines.

- **Patient data** includes sex, date of birth, and body mass index.
- **Pre-operatory data** includes the name of the surgeon, date of operation, and American Society Anesthesiologists (ASA) physical status classification system [29]. During the physician’s examination information from the patient’s medical records and the physical examination will be used to identify comorbid medical conditions (diabetes, hypertension, cardiopathy, chronic obstructive pulmonary disease, ransient ischemic attack, anticoagulant-antiplatelet therapy, and other pathologies). The Charlson comorbidity score will be calculated [29]. Preoperative grading and staging of the bladder neoplasm will be done according to standard criteria (EAU) including T-stage, G-stage, and presence of a concomitant carcinoma in situ (CIS). Preoperative therapeutic interventions, including neoadjuvant chemotherapy, palliative cystectomy, and BCG-instillation will be recorded.

- **Peri-operative data.** Data on the surgical procedure will be categorized according to the technique used for cystectomy (open / robotic-assisted / laparoscopic) as well as for urinary diversion (open / robotic-assisted / laparoscopic). Type of urinary diversion (ileal conduit / neobladder reconstruction / other) will be documented as well as whether there is a conversion to open surgery, and the reason for conversion. Total time of surgery as well as time taken for lymphadenectomy and urinary diversion (minutes) will be measured, in addition to milliliters of peri-operative bleeding. We will record multiple peri-operative data including nerve sparing (no / unilateral / bilateral), lymphadenectomy (not performed / bilateral external iliac nodes / bilateral presacral iliac), pelvic lymph node dissection (no / limited / standard / extended), partial prostatectomy, urethrectomy, and whether frozen section ureters (no / yes normal / CIS left / CIS right) or other frozen sections (no / normal urethra / CIS urethra / normal bundle) are performed.

- **Post-operative and histology data.** Post-operative staging and grading of tumors and CIS, as well as tumor histology (transitional cell carcinoma (TCC), adenocarcinoma, or other) and Gleason score [30] will be recorded. Post-operative pelvic node (total number and position) will be measured as well as the presence of prostate cancer in men. Positive margins for bladder and prostate will be measured. Number of days in hospital and readmission within 30 days post-surgery will be recoded.
- **Immediate (< 30 days) and late (30–90 days) post-operative complications.** In the event of a patient experiencing post-operative complications, the following characteristics will be recorded: number of complication events, time of first complication event, a description of each complication, Clavien-Dindo classification of immediate complications [31], and the date and description of treatment used. This information will be recorded both for immediate (30 days after surgery) and late (30–90 days after surgery) post-operative complications.

- **Follow up and outcomes.** All patients will be followed for 24 months after surgical intervention to assess multiple outcomes: mortality, tumor recurrence, continence, and sexual potency. In the event of a patient moving to another medical facility for follow-up treatment, the physician will be responsible for collecting follow-up data from them, with the patient’s consent. Follow-up data will include hospital readmission within 90 days, reoperation within 90 days, and mortality (and cause of death and autopsy data). In the event of tumor recurrence, data will be taken on date and localization of tumor recurrence, and subsequent treatment (adjuvant chemotherapy or radiation). Functional outcomes will include daytime and nighttime continence and sexual potency rates (at > 6 months and > 12 months) reported by patients to the physicians.

### Statistical analysis

Data will be cleaned and checked for discrepancies by a statistician before analysis. In cases of missing data, the responsible physician will be contacted and requested to check medical records and data-sheets for missing information. Characteristics of patients with missing data will be examined, and if necessary, sensitivity analysis or multiple imputation will be used to examine the effect of missing data on the results. Chi-square and students t-test will be used to assess differences in categorical and numerical data, respectively. The four main long-term outcomes of interest are recurrence-free survival, cancer-specific survival, overall survival, and functioning (continence and potency). Cox proportional regression models with 95% confidence intervals will be used to assess the risk of mortality or tumor recurrence according to the surgical technique used (open, laparoscopic, robot-assisted technique for cystectomy and/or urinary diversion). The exposure variable will be classified into different categories to also examine changes in surgery type during the operation (e.g., robot-assisted cystectomy and open surgery urinary diversion versus robot-assisted for both stages of the intervention). Logistic regression models, with adjustment for follow-up time, will be used to assess outcomes at different time points including: i) peri-operative factors (e.g., surgery time, blood loss, etc); ii) immediate post-operative factors (number of days in hospital post-operative complications etc) and; iii) long-term oncological and functional outcomes (mortality, tumor recurrence, continence and potency) up to 24 months post-surgery. Multivariable models will be used, with potential confounders selected both a-priori and on the basis of meeting the three statistical criteria for confounding (associated with the exposure, associated with the outcome, and not being on the causal pathway between exposure and outcome). Stratification and adjustment will be made according to the medical center, primary surgeon responsible for the operation and their years of medical experience, and various pre-surgery factors (tumor grading and staging, comorbidity, ASA score etc). Sample size is estimated according to a similar previous protocol [32], which is based on an overall complication percentage of 65% in the open radical cystectomy [33] with power set at 80% and alpha 5%, a sample size of 338 (2 × 169) patients is required to detect a decrease in the overall complication rate of 15%, i.e. from 65 to 50%.

### Role of the scientific and steering committees

The role of Steering Committee will be to supervise the planning and implementation of the registry. Specifically, they: approve the participating centers and the corresponding physician in charge of data collection; conduct quality control of the data; guide and propose relevant changes in order to meet the project objectives; analyze and revise final results to be presented at congresses and published in scientific papers. Members of the Steering Committee include: *Walter Artibani; Maurizio Brausi; Franco Gaboardi; Michele Gallucci; Giacomo Novara and; Angelo Porreca.*

The role of the Scientific Committee is to guide eventual scientific publications and/or presentation of results at conferences, and to consult with the Steering Committee. The committee will ensure that authorship eligibility guidelines are adhered to for all publications. The members include: *Alessandro Antonelli; Aldo Bocciardi; Riccardo Schiavina; Antonio Celia; Luca Cindolo; Renzo Colombo and; Andrea Minervini.*

## Discussion

### Summary

The current protocol aims to contribute additional data to the field concerning the short- and long-term outcomes of different radical cystectomy surgical techniques for patients with bladder cancer. Similar registries are ongoing, for example in the Netherlands [32].

### Limitations

The main limitation of the study design is that the treatment arms are not randomized. Clinical reasoning behind treatment choice may affect conclusions, although the extensive data collection on numerous potentially relevant factors will allow us to adjust for potential confounders. There is some value of comparative-effectiveness trials such as these, because thereare a complex range of factors and decision making by both physicians and patients that affect their choice of surgical technique, which cannot be assessed in RCTs.

### Strengths and relevance

Despite the lack of randomization, there are several advantages of the current protocol. The current literature is largely based on non-randomized, retrospective analysis of clinical data, but our protocol was designed prospectively and will include a much larger sample size than the currently available studies. The multicenter design also provides some variation to the data, with 28 clinics all across Italy providing data, thus minimizing selection bias. Multiple surgeons in multiple clinics will perform the different surgical techniques on diverse groups of patients (different regions of the country, socioeconomic groups, rural and urban population etc), which will increase generalizability of the results. Though most studies compare two surgical techniques, we will compare three types (open, laparoscopic, and robot-assisted). Another novel aspect is that we will perform analysis that accounts also for change in technique during surgery (e.g., robotic-assisted cystectomy plus open urinary diversion surgery versus robotic-assisted surgery for both parts of the operation). Importantly, we have planned a long follow-up of 24 months that will allow us to examine both the short- and long-term outcomes in patients. A large proportion of the current evidence focuses on short-term outcomes and data is sparse on patients’ functional outcomes such as continence and potency, but we have included up to two-years of follow-up on multiple factors, including survival, functioning, and oncological outcomes, thus providing a more comprehensive picture.

## Data Availability

Data sharing is not applicable to this article as no datasets were generated or analysed during the current study.

## Abbreviations

ASA: American Society Anesthesiologists
BCG: Bacillus Calmette-Guerin
CIS: Concomitant carcinoma in situ
EAU: European Association of Urology
RCT: Randomized control trial
TCC: Transitional cell carcinoma
